# C3 mesangial proliferative glomerulonephritis initially presenting with atypical hemolytic uremic syndrome: a case report

**DOI:** 10.1186/s13256-016-0992-6

**Published:** 2016-07-27

**Authors:** Can Huzmeli, Ferhan Candan, Ayse Seker, Esin Yildiz, Hatice Terzi, Mansur Kayatas

**Affiliations:** 1 Department of Nephrology, Cumhuriyet University Faculty of Medicine, Sivas, Turkey; 2 Department of Pathology, Cumhuriyet University Faculty of Medicine, Sivas, Turkey; 3 Department of Hematology, Cumhuriyet University Faculty of Medicine, Sivas, Turkey

**Keywords:** Uremia, C3 glomerulonephritis, Acute renal failure

## Abstract

**Background:**

Hemolytic uremic syndrome is characterized by acute renal failure, thrombocytopenia, and Coombs-negative hemolytic anemia. In C3 mesangial proliferative glomerulonephritis, an increase in mesangial cell proliferation without thickening in the glomerular capillary wall can be seen under light microscopy, but the definitive diagnosis is made with the immunohistologic demonstration of isolated C3 deposits in the mesangium. C3 glomerulonephritis may be detected in childhood; however, in this case report we describe the first case of isolated C3 glomerulonephritis together with atypical hemolytic uremic syndrome in an adult patient.

**Case presentation:**

Here we present a case of a 27-year-old white man with anuria who was hospitalized after being diagnosed as having hemolytic uremic syndrome accompanied by acute renal failure. Renal biopsy results revealed C3 glomerulonephritis. There was a complete recovery of renal function after hemodialysis, and prednisolone and plasma exchange treatment.

**Conclusions:**

C3 glomerulopathy is distinct from atypical hemolytic uremic syndrome although both diseases are due to abnormal control of the alternative complement pathway. In atypical hemolytic uremic syndrome activation of complement occurs on glomerular or microvascular endothelium causing a thrombotic microangiopathy; in most cases, no electron-dense deposits are seen on electron microscopy and glomerular C3 is not detected on immunofluorescence.

## Background

Advances in our understanding of the role of complement in the pathogenesis of membranoproliferative glomerulonephritis (MPGN) have led to proposed reclassification into immunoglobulin-mediated disease (driven by the classical complement pathway) and non-immunoglobulin-mediated disease (driven by the alternative complement pathway). In particular, the observation that immunofluorescence for a subset of patients with MPGN stained only for C3 without immunoglobulin, coupled with the unfolding of genetic and acquired defects in the alternative complement pathway now termed C3 glomerulopathy [1].

This is clinically characterized by non-immune hemolytic anemia, low platelet count, and organ dysfunction. The European Pediatric Research Group has divided thrombotic microangiopathies into two groups according to their etiology: thrombotic thrombocytopenic purpura (TTP) and hemolytic uremic syndrome (HUS) [2]. HUS is classically divided into the more common Shiga-like toxin-producing *Escherichia coli* HUS, which is caused by a prodromal diarrheal illness and linked to Shiga toxin-producing bacteria, and atypical HUS (aHUS), a result of a genetic defect in complement regulation [3, 4]. TTP and HUS can be difficult to differentiate due to similar clinical presentation including microangiopathic hemolytic anemia, thrombocytopenia, renal involvement, neurologic involvement, and fever. However, while neurologic manifestations are predominant in TTP, renal involvement is more prominent in HUS.

## Case presentation

A 27-year-old white man with an unremarkable medical and family history presented to our emergency department with nausea, vomiting, fever reaching 38.8°C, and bloody-mucoid diarrhea 10 to 13 times a day for the past 2 days. In that period, *Salmonella* had been found in some meat cultures in the city center of Sivas and an endemic diarrhea presenting with the same clinical manifestations had been defined. He stated that he had eaten from the meat that had previously been shown to contain *Salmonella*. His blood pressure was 120/70 mmHg and his pulse rate was 78/minute, but other physical examination findings were normal. His baseline biochemical analysis was unremarkable except for leukocytosis of 21.6×103/mm^3^. Stool microscopy demonstrated 7 to 8 red blood cells per high-power field and 1 to 2 white blood cells in some fields. No growth was detected in his blood or stool cultures. He was hospitalized in the infectious diseases clinic for gastroenteritis, and he was started on ciprofloxacin 500 mg treatment twice daily.

One week after hospitalization, his laboratory results were as follows: blood urea nitrogen 75 mg/dL, serum creatinine 10.4 mg/dL, lactate dehydrogenase (LDH) 1539 U/L, total protein 4.5 g/dL (5.7 to 8.2), and albumin 2.8 g/dL (3.2 to 4.8). His urine analysis results were normal. He was transferred to our nephrology department because he was anuric, and the possibility of HUS was under consideration. In that period, his blood pressure was 130/80 mmHg, pulse rate was 84/minute, and body temperature was 36°C; there were no pathologic findings in his physical examination. There was no growth in the specific stool culture performed for enterohemorrhagic *Escherichia coli*. Two days later, his hemoglobin (Hb) concentration dropped to 10 g/dL, direct Coombs test was negative, his reticulocyte ratio was 6 %, and there were schistocytes in his peripheral blood smear. His serologic tests were negative for anti-nuclear antibodies, cytoplasmic anti-neutrophil cytoplasmic antibodies, hepatitis B surface antigen, antibodies to hepatitis B surface antigen, antibodies to hepatitis C virus and human immunodeficiency virus, perinuclear antineutrophil cytoplasmic antibody and anti-glomerular basement membrane results; his C3 was 0.511 g/L (0.9 to 2.1) and C4 was 0.151 g/dL (0.1 to 0.4).

**Fig. 1:**
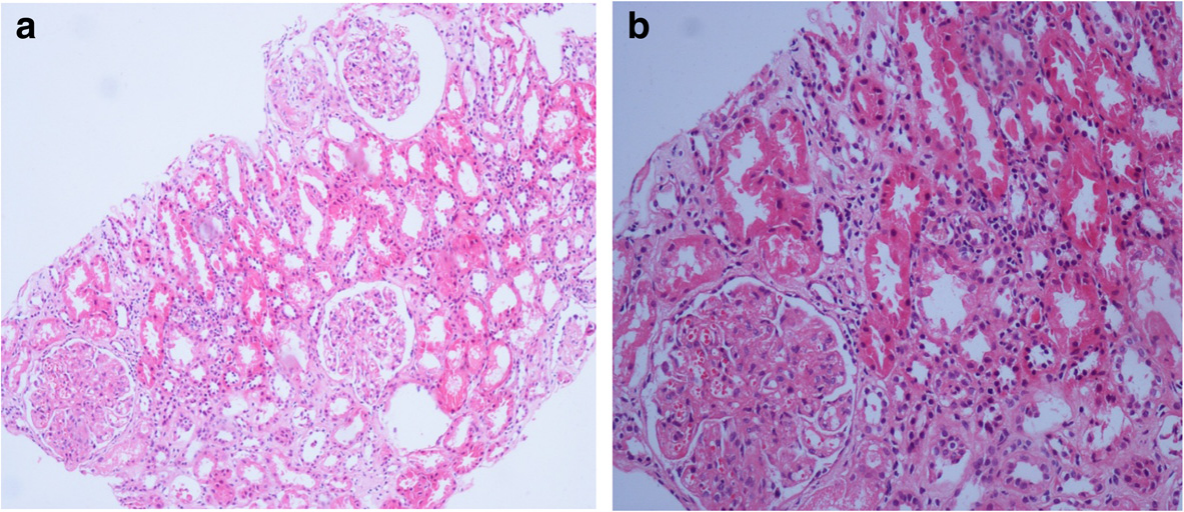
Increased mesangial matrix and mesangial cells in the glomeruli. Focal lymphocytic inflammation was observed in the interstitium. There was no pathological finding in the tubules and blood vessels. Picture on the left (**a**; HEx100); picture on the right (**b**; HEx200). *HE* hematoxylin-eosin

A urine analysis performed 2 weeks later when his urine output began to increase, revealed +4 proteinuria and normal microscopy, whereas micro-total protein in 24-hour urine was measured as 18 g/day. In addition, valsartan 160 mg/day and prednisolone 1 mg/kg/day was added to the treatment because his blood pressure increased (maximum to 170/100 mmHg), and hemodialysis and plasma exchange were commenced for aHUS plus acute renal failure. His hemodialysis therapy was discontinued after eight sessions because his creatinine levels decreased and urine output increased. Plasma exchange was performed for a total of 12 sessions until his platelet count and LDH levels returned to normal. He also received a red blood cell transfusion (two units) for symptomatic anemia (Hb 7 g/dL, hematocrit 22 %). The pathology report of the samples obtained through renal biopsy was consistent with C3 glomerulonephritis; therefore, azathioprine 100 mg/day was added to his treatment. A renal biopsy specimen stained with hematoxylin and eosin revealed six glomeruli with increased mesangial cells and matrix, and minimal focal interstitial lymphocytic inflammation. There was no pathologic finding in the tubules and blood vessels (Fig. 1). Direct immunofluorescence staining of the tissue sections revealed a total of eight glomeruli, which showed only mild to moderate mesangial granular staining for C3; no immunofluorescent staining was detected with immunoglobulin A, immunoglobulin G, immunoglobulin M, C1q, and fibrinogen. Congo red staining was negative. One month later, his serum creatinine was found to be 1.1 mg/dL, LDH was 232 mg/dL, his platelet count was 190,000/mm^3^, Hb was 12.8 g/dL, albumin was 3.5, C3 was within the normal ranges, and the micro-total protein in 24-hour urine was 1.3 g/day.

## Discussion

HUS is characterized by microangiopathic hemolytic anemia, thrombocytopenia, and renal dysfunction. In HUS, reticulocyte numbers, indirect bilirubin, and LDH levels increase as a result of intravascular hemolysis, and haptoglobin levels decrease. Fragmented red blood cells (schistocytes) and polychromasia are common in peripheral blood smears. C3 glomerulonephritis is recognized by the presence of glomerulonephritis under light microscopy, immunofluorescent staining with C3, but not with immunoglobulins, C4 or C1q, and the presence of mesangial or subendothelial deposition, which can be observed using electron microscopy [5–7]. C3 glomerulonephritis results from deposition of C3 degradation products and terminal complement components in glomeruli that result from the activation of alternative complement pathway due to the defects of complement-regulating proteins. The immunohistologic diagnosis of C3 glomerulonephritis is made based on the presence of mesangial C3 deposition together with the absence of immunoglobulin and other complement components [5]. Mesangial C3 deposition is seen in primary and secondary glomerulonephritis and in collagen diseases. Clinically isolated hematuria appears in various forms, ranging from normal renal function to end-stage renal insufficiency. On pathological examination, it progresses with mild glomerular abnormalities to various degrees of mesangial cell proliferation and may be accompanied by glomerulosclerosis. The clinical and laboratory findings of our patient were not suggestive of autoimmune diseases, such as systemic lupus erythematosus, or malignant diseases. The presence of hypertension, heavy proteinuria, renal dysfunction, severe mesangial proliferation, sclerotic glomeruli, interstitial fibrosis, tubular atrophy, and resistance to steroid therapy are indicators of poor prognosis in C3 glomerulonephritis. Our patient had renal dysfunction, hypertension, and heavy proteinuria as indicators of poor prognosis. Glomerulonephritis has been anecdotally reported in association with HUS.

Different types of glomerulopathies (membranous glomerulonephritis, focal segmental glomerulosclerosis, MPGN, immunoglobulin A nephropathy, C1q nephropathy, and C3 glomerulonephritis) can be complicated by HUS. Boyer *et al*. [8] reported two cases of HUS associated with complement factor H (CFH) deficiency in native kidneys, and glomerulonephritis with isolated C3 deposits after renal transplantation [9–13]. Genetic or acquired C3 nephritic factor (C3NeF) abnormalities of complement-regulating proteins are rarely associated with C3 deposition. CFH plays a regulatory role in the alternative complement pathway and its dysfunction may lead to many renal diseases, such as HUS and C3 glomerulonephritis. Factor H, factor I, membrane cofactor protein (MCP), and C3NeF were studied in a study conducted on patients with isolated C3 deposition without HUS. The patients were divided into two groups: the first group consisted of patients with MPGN type 1 and C3 deposition (*n*=13), and the second group comprised patients with C3 glomerulonephritis with non-MPGN C3 deposition (*n*=6). In group 1, C3NeF mutation was detected in five patients and was indefinite in one, *CFH* mutation was detected in one patient, and *MCP* mutation was detected in one patient. In group 2, C3NeF mutation was detected in two patients and was indefinite in one, *CFH* mutation was detected in two patients, and *complement factor I* mutation was detected in two patients. It was emphasized that patients with non-MPGN type 1, that is, those with C3 glomerulonephritis, and patients with HUS, share common genetic risk factors; a relation was determined between the regulation of alternative pathway and genetic abnormalities in 70 % of the patients [6].

## Conclusions

In conclusion, glomerulonephritis diseases, particularly those that coexist with isolated C3 glomerulonephritis and aHUS, may be associated with *CFH*, *complement factor I*, and *MCP* mutations. These mutations have been demonstrated in both diseases. In addition, complement mutations in other cases of glomerulonephritis have been published as case reports. Improved understanding of MPGN and, in particular, the activity of the alternative complement pathway in C3 glomerulopathies will lead to improved outcomes with targeted therapy in these patients.

## Abbreviations

aHUS, atypical hemolytic uremic syndrome; C3NeF, C3 nephritic factor; CFH, complement factor H; Hb, hemoglobin; HUS, hemolytic uremic syndrome; LDH, lactate dehydrogenase; MCP, membrane cofactor protein; MPGN, membranoproliferative glomerulonephritis; TTP, thrombotic thrombocytopenic purpura.
